# Proximate composition determination in goat cheese whey by near infrared spectroscopy (NIRS)

**DOI:** 10.7717/peerj.8619

**Published:** 2020-02-12

**Authors:** Isadora Kaline Camelo Pires de Oliveira Galdino, Hévila Oliveira Salles, Karina Maria Olbrich dos Santos, Germano Veras, Flávia Carolina Alonso Buriti

**Affiliations:** 1 Centro de Ciências Biológicas e da Saúde, Universidade Estadual da Paraíba, Campina Grande, Paraíba, Brazil; 2 Embrapa Caprinos e Ovinos, Empresa Brasileira de Pesquisa Agropecuária, Sobral, Ceará, Brazil; 3 Embrapa Agroindústria de Alimentos, Empresa Brasileira de Pesquisa Agropecuária, Rio de Janeiro, Rio de Janeiro, Brazil; 4 Centro de Ciências e Tecnologia, Universidade Estadual da Paraíba, Campina Grande, Paraíba, Brazil

**Keywords:** Food analysis, Dairy, Chemometric analysis, By-product upgrading, Seasonal composition

## Abstract

**Background:**

In Brazil, over the last few years there has been an increase in the production and consumption of goat cheeses. In addition, there was also a demand to create options to use the whey extracted during the production of cheeses. Whey can be used as an ingredient in the development of many products. Therefore, knowing its composition is a matter of utmost importance, considering that the reference methods of food analysis require time, trained labor and expensive reagents for its execution.

**Methods:**

Goat whey samples produced in winter and summer were submitted to proximate composition analysis (moisture, total solids, ashes, proteins, fat and carbohydrates by difference) using reference methods and near infrared spectroscopy (NIRS). The spectral data was preprocessed by baseline correction and the Savitzky–Golay derivative. The models were built using Partial Least Square Regression (PLSR) with raw and preprocessed data for each dependent variable (proximate composition parameter).

**Results:**

The average whey composition values obtained using the referenced methods were in accordance with the consulted literature. The composition did not differ significantly (*p* > 0.05) between the summer and winter whey samples. The PLSR models were made available using the following figures of merit: coefficients of determination of the calibration and prediction models (*R*^2^cal and *R*^2^pred, respectively) and the Root Mean Squared Error Calibration and Prediction (RMSEC and RMSEP, respectively). The best models used raw data for fat and protein determinations and the values obtained by NIRS for both parameters were consistent with their referenced methods. Consequently, NIRS can be used to determine fat and protein in goat whey.

## Introduction

In Brazil, goat cheese production occurs on small to medium-sized dairy processing plants (Buriti et al., 2014; Pereira et al., 2019), since Brazilian goat breeding is an activity performed mainly by small producers (Facó et al., 2011). The liquid whey obtained as a result of cheesemaking is a strong pollutant and is often discarded as an effluent without treatment (Buriti et al., 2014; Guimarães, Teixeira & Domingues, 2010; Pereira et al., 2019).

Goat milk production in Brazil was estimated at 25.353 tons in 2017 (Instituto Brasileiro de Geografia e Estatística, 2017). Considering that each kilo of cheese releases nine liters of whey during processing and the hypothesis that 50% of Brazilian goat milk production was used in cheese production (Tranjan et al., 2009), approximately 11.400 tons of whey was generated. The oxygen demand of whey discarded as an effluent is between 500.000 and 800.000 mg/L (Tranjan et al., 2009; Barukcic, 2018).

Goat whey has a cloudy aspect, with its color varying from green to yellow, and it also has a fresh, slightly sweet or acidic taste. It contains approximately 55% of milk nutrients: soluble proteins, lactose, vitamins, minerals and a minimum amount of fat (Guimarães, Teixeira & Domingues, 2010). Considering this and the necessity of avoiding environmental impacts related to improper whey disposal, it leads the scientific community to search for viable alternatives for the use of goat whey. According to Almeida, Tamine & Oliveira (2008), the simplest and most economic process to use whey, for example, is to return it to the processing line while it’s still a fluid, thus the byproduct can be used in the formulation of dairy-based beverages and desserts (Almeida Neta et al., 2018; Pereira et al., 2019).

Milk composition and technological properties of processed milk can significantly influence the yield of cheese, nutrients recovery in curd (Pazzola et al., 2019; Vacca et al., 2018), and consequently the composition of whey. Milk composition and coagulation properties may vary among different goat breeds (Pazzola et al., 2019) and can differ according to the season (Ozrenk & Inci, 2008), thus affecting cheesemaking and the resultant whey (Garcia, Puerto & Baquero, 2006). The precise composition of the whey is relevant to attend the regulatory standards required for whey derived products such as dairy beverages (Brasil, 2005). Therefore, there are several well-established methods for physicochemical determinations, including those officially adopted by the current Brazilian legislation (Brasil, 2018). However, these methods require time, trained manpower and expensive reagents in order to be carried out.

For that reason, it is necessary to search for analytical methods with fast, reliable, waste-free and simple analysis technologies to facilitate the quality evaluation of milk and whey. In this context, near infrared spectroscopy (NIRS) has become the target of continuous study because of its nondestructive technique that allows the analysis of samples to be done without previous treatment and waste production. This method also reduces time and the need of qualified personnel (Silva et al., 2012). Consequently, NIRS offers several advantages for food quality control in comparison to a traditional methodology (Costa et al., 2016), including the ones used in dairy products (Stocco et al., 2019).

Some studies were carried out with NIRS, focusing on the quality control of whey in some processing steps such as heat treatment, filtering and hydrolysis (Kucheryavskiy & Lomborg, 2015; Pouliot et al., 1997). Nonetheless, more studies of the composition of crude whey are still required, particularly concerning the possible seasonal variation.

The main objective of this study was to determine the proximate composition of different batches of goat whey from cheeses produced in different seasons and to evaluate the use of NIRS as an alternative method to obtain the compositional parameters of these samples.

## Materials and Methods

### Obtaining crude whey

Milk for cheese manufacturing was produced from Saanen goats at Embrapa Goats and Sheep, located in Sobral, Ceará, Brazil. Twenty four batches of whey from goat *Coalho* cheese, produced as described by Egito & Laguna (1999) using chymosin from *Aspergillus niger* var. *awamori* (Ha-la^®^ coagulant; Chr. Hansen, Valinhos, Brazil) and a mesophilic homofermentative culture of *Lactococcus lactis* ssp. *lactis* and *L. lactis* ssp. *cremoris* (R-704 lactic culture; Chr. Hansen, Valinhos, Brazil), were supplied by Embrapa. Twelve batches (2, 3, 4, 5, 6, 7, 14, 15, 16, 17, 18 and 24) were produced during spring/summer (October–March), 11 batches (1, 8, 9, 10, 11, 12, 13, 19, 20, 21 and 22) were produced during autumn/winter (April–September) and one of the batches has an undetermined production. Within these batches, 17 mixtures (1/2, 1/3, 3/4, 4/5, 5/6, 6/7, 7/8, 8/9, 9/10, 10/11, 11/12, 13/14, 15/16, 17/18, 19/20, 21/22 and 23/24) were made without following seasons of the year, in the proportion of 1:1 to obtain a total of 41 samples of goat cheese whey.

### Analysis of total solids, moisture, ashes, fat, protein and carbohydrate by difference

The analysis of total solids and moisture content were obtained by drying 2 g of a sample in a Quimis vacuum oven (model 0819V2; Diadema, São Paulo, Brazil) at 70 °C (Instituto Adolfo Lutz, 2008). The ash content was obtained by the incineration of 2 g of a sample at 550 °C until the total elimination of organic matter (Instituto Adolfo Lutz, 2008). The fat content was obtained by the Gerber method (Instituto Adolfo Lutz, 2008). Protein content was estimated from the analysis of the nitrogen content through the Kjeldahl micro method, using a conversion factor of 6.38 for milk and dairy products (AOAC, 2003). The total carbohydrate content was calculated by difference to obtain 100% of the total composition (Food & Agriculture Organization of the United Nations, 2003). All parameters were obtained in duplicate samples.

### Spectroscopic analysis

All spectra were recorded in triplicate using a PerkinElmer 750 Lambda spectrometer (PerkinElmer, Waltham, MA, USA) equipped with 1 cm optical path quartz cell, tungsten source and a R928 photomultiplier tube and Peltier-cooled PBS detection system, in the wavelength region between 700 and 1,800 nm, with a resolution of 1 nm. The equipment used was calibrated.

### Chemometric analysis

The spectral data was analyzed for all whey batches and their mixtures (*n* = 41) with the Unscrambler 9.7 software (Camo Software, Oslo, Norway), using the Partial Least Square Regression (PLSR) of proximate composition analysis (moisture, total solids, ash, protein, fat and total carbohydrates). The raw data of these measurements are provided in the Supplemental File 1. Different types of pre-processing data were tested, including baseline correction, multiple scattering correction (MSC) and Savitzky & Golay (1964) smoothing with 1st derivative. The root mean square error calibration and prediction (RMSEC and RMSEP respectively), *R*^2^ and systematic error (bias) values were analyzed.

### Statistical analysis

The results of moisture, total solids, ash, protein, fat and total carbohydrate by difference were compared and the response variables were expressed as mean ± standard deviation: samples 1–13 in triplicates (*n* = 39), samples 14–41 in duplicates (*n* = 56), for a total of *n* = 95. The different batches of whey from goat cheese with determined season of production (*n* = 23) were grouped according to the production period being classified as autumn–winter (whey obtained from cheeses manufactured from April to September) and spring–summer (whey obtained from cheeses manufactured from October to March). The raw data of all these measurements are also provided in the Supplemental File 1. The normality of these results was evaluated through the Shapiro–Wilks and Kolmogoroy–Smirnov tests, with α 0.05. Since the normality of the results was not confirmed, a non-parametric analysis was performed with the Mann Whitney *U* test to identify the contrasts between winter and summer whey samples (Bower, 1997), using the Statistica software version 6.0 (Statsoft Inc., Tulsa, OK, USA).

## Results

### Proximate composition

The mean values of the goat cheese whey proximate composition, obtained by conventional methods, were 93.49% moisture; 6.51% total solids; 0.63% ash; 1.19% protein; 0.46% fat and 4.24% carbohydrates by difference (Table 1).

**Table 1:** Proximate composition of the overall goat cheese whey samples and their mixtures and of the goat cheese whey divided for the spring–summer^1^ and autumn–winter^2^ seasons (mean ± standard deviation).

Parameters	Overall samples	Spring/summer	Autumn/winter
Moisture (%)	93.53 + 0.68	93.50 ± 0.88^a^	93.47 ± 0.47^a^
Total solids (%)	6.47 + 0.68	6.50 ± 0.88^a^	6.53 ± 0.47^a^
Ash (%)	0.62 + 0.13	0.60 ± 0.21^a^	0.66 ± 0.079^a^
Protein (%)	1.22 + 0.21	1.22 ± 0.20^a^	1.16 ± 0.17^a^
Fat (%)	0.45 + 0.14	0.45 ± 0.17^a^	0.48 ± 0.16^a^
Carbohydrate by difference (%)	4.18 + 0.16	4.23 ± 0.44^a^	4.23 ± 0.79^a^

**Notes:**

*n* = 41 samples.

1 Samples produced between October and March (*n* = 12).

2 Samples produced between April and September (*n* = 11).

a Letters in the same line do not differ significantly for the same parameter between samples of spring/summer and autumn/winter seasons.

In Table 1 the proximate composition of goat cheese whey produced in the different seasons of the year is also shown. There was no significant difference, *p* < 0.005, in the proximate composition between spring/summer and autumn/winter seasons.

### NIR spectra

**Figure 1:**
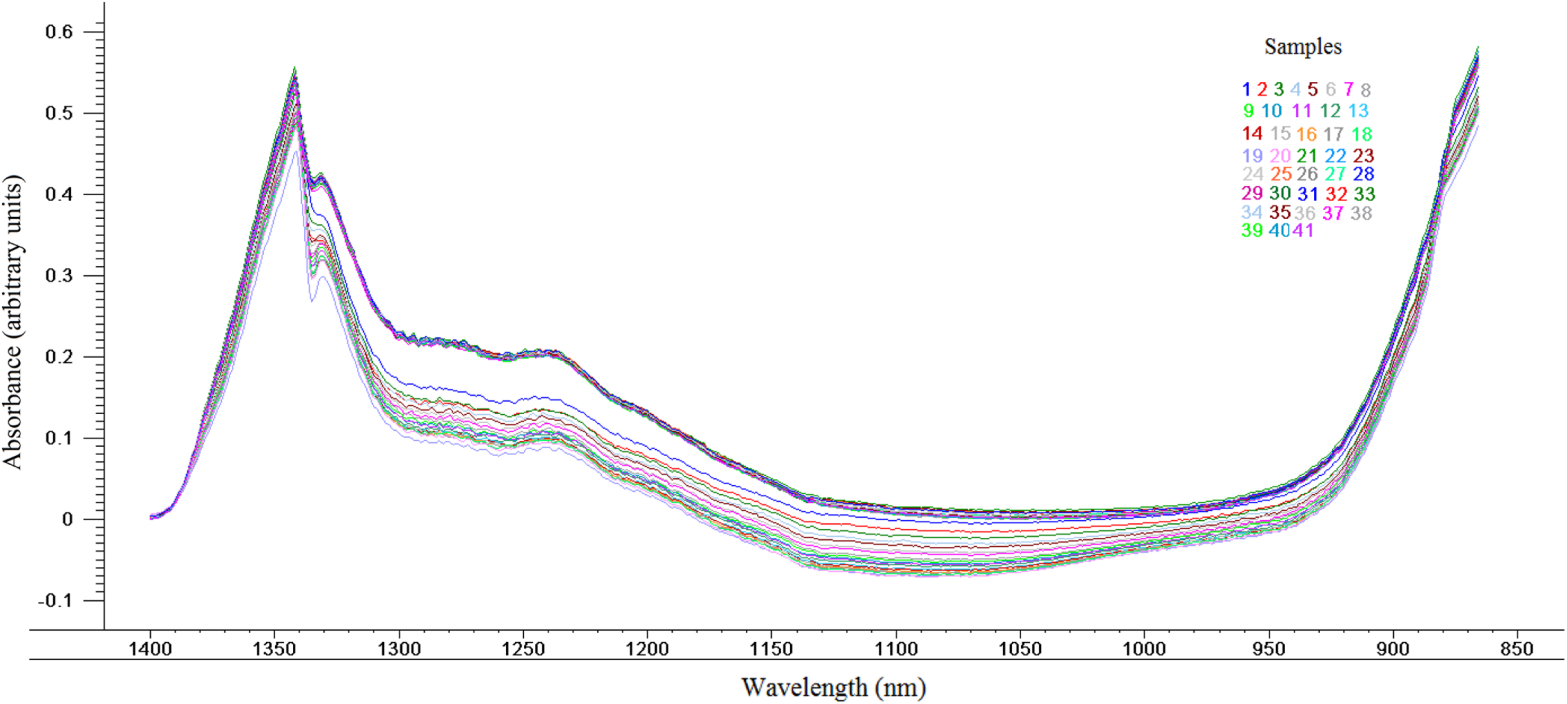
Near infrared spectra between 866 and 1400 nm obtained for the different batches whey of goat cheese (*n* = 41).

Spectra was obtained from spectral portions between 700 and 1,800 nm. To improve data analysis and chemometric models, spectral portions between 700 and 865 nm and between 1,401 and 1,800 nm were not considered, those being the noisy regions. Therefore, the region of the spectrum used was from 866 to 1,400 nm, according to Fig. 1, thus containing more relevant information concerning the proximate composition of the goat cheese whey.

### Chemometric models

The PLS calibration models were built using the mean values of compositional analysis (moisture, total solids, ash, protein, fat and carbohydrate) of each of the 41 samples. Thirty-one samples (75%) were used for the calibration step and 10 samples (25%) were used for the prediction (validation) step of the model using sample set partitioning based on joint X- and Y-blocks sample variable after pre-processing (Galvão et al., 2005).

### Calibration and validation model

The pre-processing used was a multiplicative scatter correction (MSC), smoothing baseline removal and Savitzky & Golay (1964), first derivatives, 11-points window and width and 2nd order polynomial, with the purpose of reducing and correcting possible interferences related to scattering, baseline shift, path-length variation and overlapping bands. The accuracy of the models was expressed as the RMSEC and RMSEP, *R*^2^ and error systematic (bias).

The results of PLS calibration and validation models of the proximate composition of goat cheese whey samples are shown in Table 2.

**Table 2:** Results of PLS calibration and validation models of proximate composition of goat cheese whey samples.

Component	Pretreatment	Calibration			Validation		
		*R*^2^	RMSEC	BIAS	*R*^2^	RMSEP	BIAS
Moisture	No treatment	0.29	0.61	−3.69 × 10^−6^	0.39	0.37	0.44
	Baseline	0.33	0.51	−2.95 × 10^−6^	0.14	0.73	−0.33
	SG1211	0.30	0.60	−3.94 × 10^−6^	0.41	0.36	0.07
Total solids	No treatment	0.99	0.05	−2.31 × 10^−7^	0.31	0.66	0.43
	Baseline	0.43	0.54	5.08 × 10^−7^	0.54	0.32	−0.18
	SG1211	0.33	0.50	1.54 × 10^−7^	0.069	0.78	0.03
Ash	No treatment	0.18	0.10	2.40 × 10^−8^	0.24	0.13	−0.03
	Baseline	0.16	0.10	1.83 × 10^−8^	0.17	0.13	−0.02
	SG1211	0.26	0.09	2.21 × 10^−8^	0.21	0.13	0.03
Carbohydrate	No treatment	0.24	0.60	−1.92 × 10^−7^	0.37	0.33	−0.17
	Baseline	0.24	0.60	3.23 × 10^−7^	0.30	0.35	−0.21
	SG1211	0.28	0.58	−2.23 × 10^−7^	0.38	0.33	−0.17
Fat	No treatment	0.99	0.01	−1.13 × 10^−7^	0.64	0.07	−0.02
	Baseline	0.99	0.01	−1.59 × 10^−7^	0.63	0.07	−0.02
	SG1211	0.99	0.01	−3.51 × 10^−8^	0.44	0.08	−0.03
Protein	No treatment	0.63	0.11	9.23 × 10^−8^	0.56	0.15	−0.02
	Baseline	0.70	0.10	2.52 × 10^−7^	0.42	0.17	0.07
	SG1211	0.77	0.09	1.54 × 10^−8^	0.29	0.19	0.06

As observed in Table 2, the preprocessing used was not adequate to significantly improve the models and therefore the raw data was used following the principle of parsimony.

The RMSEC and RMSEP values for moisture were 0.61 and 0.37 respectively. The *R*^2^ values for calibration and prediction in all treatments were considered low for the constructions of the model.

In the model developed for calibration of total solids, an *R*^2^ of 0.99 and a RMSEC of 0.048 were obtained, and for validation an *R*^2^ of 0.31 and a RMSEP of 0.66 were obtained.

The results obtained for the ash content in the first Savitzky–Golay derivative were *R*^2^ of 0.26 for calibration and 0.21 for validation.

For the carbohydrate calibration and validation models, *R*^2^ was obtained from 0.24 to 0.37, respectively. The RMSEC and RMSEP values were 0.60 and 0.33, respectively.

The best models were obtained for fat and protein. The fat model was developed with *R*^2^ calibration of 0.99, RMSEC of 0.01, *R*^2^ validation of 0.64 and RMSEP of 0.07. For protein, the model obtained *R*^2^ calibration of 0.63, RMSEC of 0.11, *R*^2^ validation of 0.56 and RMSEP of 0.15.

The relative errors between the reference method and the NIR for fat and protein were 0.97% and 0.81% respectively (Table 3).

**Table 3:** Content (%) of fat and protein by the reference method and NIR and their relative errors (%).

Content	Methods	Mean	Min	Max	Relative error
Fat (%)	Reference	0.45	0.20	0.83	0.97
	NIR	0.44	0.187	0.82	
Protein (%)	Reference	1.22	0.46	1.58	0.81
	NIR	1.23	0.78	1.47	

Observing the value of the relative error, it can be inferred that the constructed model can predict, with reduced errors, the fat and protein contents, in which an excellent correlation between both methods can be observed.

## Discussion

The values of proximate composition obtained in the present study were close to those mentioned in the literature. Most studies also reported moisture values in goat whey very close to 93% (Borba et al., 2014; Chaves de Lima, De Moura Fernandes & Cardarelli, 2017), total solids of approximately 7% (Gomes et al., 2013; Sanmartín et al., 2012), ash content around 65% (Borba et al., 2014; Gomes et al., 2013; Chaves de Lima, De Moura Fernandes & Cardarelli, 2017; Sanmartín et al., 2012), protein content between 0.84% and 1.40% (Borba et al., 2014; Chaves de Lima, De Moura Fernandes & Cardarelli, 2017), fat content of 0.8% or lesser (Borba et al., 2014; Chaves de Lima, De Moura Fernandes & Cardarelli, 2017), and carbohydrate content in the range of 3–5% (Borba et al., 2014; Chaves de Lima, De Moura Fernandes & Cardarelli, 2017). According to Garcia, Puerto & Baquero (2006), variations in the composition of goat whey nutrients, particularly for protein and carbohydrates, depend on the characteristics of the milk among other factors, and the type of cheese produced. Although whey in its crude and fluid form has a low percentage of proteins, it has a high biological value which is excellent for metabolic efficiency and the ability to fix calcium. Moreover, goat whey proteins contain, in adequate amount and proportion, all essential amino acids required for human consumption (Barukcic, 2018).

No significant difference was observed in all the analyzed parameters (*p* > 0.05) between the seasons of the year in which the whey was produced. This result differed from the one reported by Lievore et al. (2015) in which they assessed the proximate composition of the acid whey from the Petit Suisse cheese production and established that all analyzed parameters, except lactose, differed significantly during the year.

Despite the lack of seasonal differences in the composition of goat whey of the present study, it is reported that breed, animal feed, farming systems (particularly the extensive system), lactation and milking usually affect the amount and quality of milk used for cheese production, which are related to the seasonal changes that can occur in the composition of cheese whey (Ferro, Tedeschi & Atzori, 2017; Šlyžius, Šlyžienė & Lindžiūtė, 2017). Therefore, it is possible to assume that the animals used in milk production (Saanen goats), that produced the resulting whey for this study, were most likely receiving the same amount of nutrients during the summer and winter months.

For construction of the model for moisture analysis by NIRS, the *R*^2^ values for calibration and validation were considered low in all treatments. Madalozzo, Sauer & Nagata (2015), in their study on the physicochemical characterization of ricotta cheese also using NIRS methodology, verified a correlation coefficient of 0.851% for calibration and a coefficient of 0.757% for validation. They also stated that there was a lower capacity prediction model for moisture determination as verified in the present study. On the other hand, Nagarajan, Singh & Mehrotra (2006) obtained *R*^2^ values for calibration validation of 0.9942 and 0.9822, respectively.

Sultaneh & Rohm (2007) evaluated the total solids content in samples of non-homogenized and homogenized curd, obtaining satisfactory results with *R*^2^ of 0.994 and 0.997 respectively. Dracková et al. (2008) analyzing goat milk by NIRS obtained *R*^2^ results of calibration and validation for total solids of 0.940 and 0.899 respectively.

Barabássy (2001) obtained *R*^2^ of 0.99 at various wavelengths for ashes in his study about the application of the technique of NIRS to nondestructive investigation of mixed dairy products.

Dracková et al. (2008), using NIRS for determination of fat in goat milk, obtained values of *R*^2^ 0.951 for calibration and *R*^2^ 0.924 for validation, these values differ from the ones presented in this study, which were 0.99 for calibration and 0.64 for validation.

Observing the relative error value, it can be inferred that the model constructed can predict, with reduced errors, the fat contents, in which an excellent correlation between the two methods can be observed. Whey is recognized as an ingredient with reduced fat content and, therefore, can be used to produce low fat products. Kucheryavskiy & Lomborg (2015), while evaluating the whey composition during the filtering process using NIRS and competitive adaptive reweighted sampling, were able to predict the fat content (*R*^2^ = 0.996), besides the total solids (*R*^2^ = 0.999) and protein amount (*R*^2^ = 0.999), with high accuracy and precision. Consequently, NIR would facilitate this quantification, also reducing the time of analysis and increasing whey processing by the industry.

Inácio, De Moura & De Lima (2011), using chemometric tools for the classification of milk powder samples and quantification of proteins, obtained satisfactory data prediction values with 1st derivative in a 15-point window with *R*^2^ equal to 0.98% and RMSEP equal to 0.52. High coefficient for proteins was also obtained by Sustová, Ruzicková & Kuchtík (2007), with a *R*^2^ of 0.989. These authors confirmed that NIRS allows easy and rapid control of milk composition, with analysis in-situ and in-line and it may improve the economic development of small dairy producers, such as cheese makers.

Water represents about 93–94% of the constituents of the whey. This component presents an intense absorption band of infrared radiation. The intensity of the water absorption band causes it to overlap the characteristic adsorption bands of the proteins (Etzion et al., 2004). It can generate altered results for the analysis of proteins from NIRS in comparison to that obtained from fat, with the best adjusted model in this article.

According to Trindade et al. (2019) dairy beverages is the largest category of dairy products manufactured using whey in the Brazilian industry. The Technical Regulation on the Identity and Quality of Dairy Beverages (Brasil, 2005) establishes the minimum amount of dairy protein in these products. For example, dairy beverages with addition of ingredients other than those of dairy origin must have minimum of 1 g/100 g and dairy beverages without addition of such ingredients must have a minimum of 1.7 g/100 g. Therefore, the immediate quantification of these proteins would facilitate the rapid and appropriate use of the fluid whey in the dairy beverage production process. Thus, knowing the amount of protein, makes it possible to know how much whey is required to be added to the product so it is in accordance with the legislation regarding the content and the most appropriate type of beverage to be prepared with whey, considering that the requirement of protein concentration differs for each category of dairy beverage.

In contrast, the carbohydrate content *R*^2^ was considered low in all treatments and consequently, not suitable for the construction of a good model. This is possibly related to the fact that the data obtained for this nutrient had been an estimate from other components of the whey. The determination of the carbohydrate content used in this study was not an analytical measure. According to Food & Agriculture Organization of the United Nations (2003) other compounds could interfere in the estimate of the total carbohydrates, such as organic acids like lactic acid, and possibly because of this fact the model did not present a good prediction for this parameter. Similarly to the one verified in the present study, Kucheryavskiy & Lomborg (2015) were unable to obtain a good model for the lactose content of filtered whey, indicating the challenge of estimating this nutrient on the byproduct.

## Conclusions

The analytical values obtained by reference method for the different parameters analyzed were generally close to those found in literature.

No significant differences were found between the composition of the whey produced in the summer and winter seasons for all parameters analyzed.

The models obtained from the NIRS analysis with the highest adjustment were achieved without data treatment to determine the percentage of fat and proteins. The root mean squared error calibration and prediction (RMSEC and RMSEP, respectively), *R*^2^ and systematic error (bias) values were analyzed. However, it was observed that the preprocessing did not contribute significantly to the improvement of the predictive capacity on models, thus resulting in models being constructed without any pretreatment. The preprocessing method used was not adequate to significantly improve the models thus, raw data was used under the principle of parsimony.

The values obtained in the determination of the fat and protein contents by NIRS were consistent based on the reference method, with the mean error between both methods being 0.97% and 0.81%, respectively, indicating a good performance for the determination of these nutrients using PLS. These results demonstrate a potential use of NIRS for the determination of composition in samples of goat whey with reduced time and less expense with personnel, samples and reagents. It aims the fast targeting for using this byproduct to its most suitable applications according to nutrient content, avoiding its improper discharge and environmental pollution.

This study also opens new possibilities for further studies also related to the use of NIRS for the evaluation of properties of whey, such as the prediction of pH values and titratable acidity, which are important aspects for the technological re-usage of whey, complimentarily to the proximate composition evaluated in the present study.

## Supplemental Information

10.7717/peerj.8619/supp-1 Supplemental Information 1 Raw data of the proximate composition and near infrared spectroscopy results.

## Additional Information and Declarations

### Data Availability

The following information was supplied regarding data availability: The raw measurements are all available in the Supplemental Files.
